# Knowledge and Attitude of Aseer Region Pharmacists Toward Biosimilar Medicines: A Descriptive Study

**DOI:** 10.3390/healthcare13243295

**Published:** 2025-12-15

**Authors:** Saeed Alqahtani, Mona Almanasef

**Affiliations:** 1 Pharmacy Department, Khamis Mushyte General Hospital, Khamis Mushyte 62433, Saudi Arabia; saeedalqahtani34@gmail.com; 2 College of Pharmacy, King Khalid University, Abha 62529, Saudi Arabia; 3 Department of Clinical Pharmacy, College of Pharmacy, King Khalid University, Abha 62529, Saudi Arabia

**Keywords:** biosimilars, biosimilar medicines, drugs, pharmacists, health care staff, knowledge, attitude, Saudi Arabia

## Abstract

Background: Many biological drugs have a rival version produced from different cell lines by other manufacturers; these drugs are referred to as biosimilars. By providing accurate information, encouraging patient and medical community acceptance, and advocating for their appropriate usage, pharmacists can play a crucial role in supporting the uptake of biosimilar medicines. Aim: This study aimed to assess pharmacists’ knowledge and attitudes toward biosimilar medicines in the Aseer region in Saudi Arabia. Methods: The study employed a descriptive, cross-sectional design using an anonymous online self-administered questionnaire. The questionnaire was developed by adapting a previously validated instrument and consisted of three sections: demographic data, knowledge about biosimilars, and attitudes toward biosimilars. Two non-probability sampling approaches, i.e., convenience and snowball sampling, were using for data collection. Results: A total of 298 pharmacists participated in the current study. Overall, a total of 135 (45.3%) demonstrated good knowledge of biosimilar medicines, while 163 (54.7%) exhibited poor knowledge. The median knowledge score among the study participants was 5 (5–6). Only 26.2% of pharmacists in the current study correctly identified that biosimilars were not generics and not interchangeable with reference biologics. More experienced pharmacists and those working in industry-related sectors demonstrated greater knowledge of biosimilars (*p* < 0.05). Pharmacists in the current study demonstrated generally favorable attitudes toward biosimilar medicines. Conclusions: The current study revealed knowledge gaps regarding biosimilar medicines among pharmacists. Targeted educational initiatives, continuing professional development opportunities, and enhanced curricular content could be implemented to address these gaps.

## 1. Introduction

Biotechnology-produced biologics are sophisticated drugs that contain active components generated from living cells or organisms. They are created using recombinant DNA technology and complex cellular system [1]. As a result of the high cost of branded biologics, biosimilars have become an attractive and affordable treatment option for many debilitating and potentially fatal diseases, including autoimmune disorders (e.g., rheumatoid arthritis, multiple sclerosis, severe psoriasis), cancer, diabetes, and other rare or life-threatening disorders [2,3]. The World Health Organization (WHO) defined a biosimilar product as a biotherapeutic product that exhibits comparable quality, safety, and efficacy to an already licensed reference biotherapeutic product [4,5,6]. However, biosimilars cannot precisely imitate the original reference product due to their inherent heterogeneity, high molecular weight, batch-to-batch variability, and complex structure [7,8]. Biosimilar medicines differ from traditional generics and require more rigorous standards for assessing their quality, efficacy, and safety compared to generic medications [9,10,11]. The process of approving biosimilars is subject to stringent regulation. 

The authorization criteria outlined by the United States Food and Drug Administration (FDA) and the European Medicines Agency (EMA) guidelines necessitate completing equivalence procedures. These procedures are designed to establish robust comparability between the biosimilar and the reference biologic, ensuring the absence of any clinically significant disparities in quality, safety, and efficacy [1,12,13]. Unlike traditional generic medications, which can be “substituted” at the pharmacy level without prescriber consent, not all biosimilars are considered “interchangeable” with their reference products [13,14,15]. This means that substitution generally requires the intervention or approval of the prescribing clinician [13,15]. To obtain an interchangeable designation, a biosimilar must meet specific FDA criteria in addition to fulfilling the standard requirements for biosimilarity [14].

Pharmacists, as drug experts, must maintain accurate knowledge and a positive perception of this emerging category of medications to ensure the safe and effective utilization of biosimilars. To further their education and stay current, pharmacists should take the initiative to learn about and stay updated on the most recent medical literature [16]. Because biosimilars are becoming more widely available, pharmacists in hospital and community settings must advise their patients about these medications. Previous published research conducted across various countries generally showed positive attitudes toward biosimilars; however, knowledge gaps were observed concerning different aspects of biosimilars, which necessitates the need for targeted interventions [8,17,18,19]. Considering this, evaluating the pharmacists’ knowledge and understanding of biosimilars is essential. Accordingly, the primary aim of the present study was to assess the knowledge of biosimilar medications among pharmacists in the Aseer region and identify factors that may influence their knowledge and expertise. Strengthening such knowledge is crucial for enhancing treatment efficacy for patients receiving these novel medications. A secondary aim was to explore pharmacists’ attitudes towards biosimilar medicines, providing a more comprehensive understanding of their perspectives.

## 2. Methodology

### 2.1. Study Design and Setting

The research design employed in this study is a descriptive cross-sectional approach using an online anonymous self-administered questionnaire. The research was conducted in the Aseer region, located in southern Saudi Arabia, from January 2024 to February 2024. The Aseer region was selected because the research team is based in this area, which provided practical convenience and direct access to a diverse group of practicing pharmacists.

### 2.2. Study Participants

Eligible participants included licensed pharmacists working across various pharmacy sectors in the Aseer region of Saudi Arabia. According to data from the Investment Administration in Aseer Health, the study population includes 1257 licensed community pharmacists and 601 hospital pharmacists in the Aseer region. After excluding pharmacists who did not work directly in pharmacies or medical supplies, the final target population consisted of 1588 pharmacists. The recommended sample size was 310 pharmacists, calculated using the Raosoft^®^ online sample size calculator (Raosoft Inc., Seattle, WA, USA) with a 5% margin of error, a 95% confidence level, and a 50% response distribution.

### 2.3. Questionnaire

The questionnaire was developed by adapting a previously validated instrument for which copyright permission was obtained [17]. The original tool was selected because it aligned closely with the aims of the current study and demonstrated acceptable internal consistency, with Cronbach’s alpha values above 0.7 across all domains in the original study [17]. For the present research, the demographic section was newly developed by the researchers to suit the study context, while the knowledge and attitudes sections were taken directly from Abusara et al. (2023) without modifying the original phrasing, except for the removal of the final item in the attitude domain, i.e., “In my opinion, pharmacist should be allowed to substitute a biologic reference drug with a biosimilar drug after patient agreement”, as this item addresses a policy-level regulatory issue rather than an attitudinal construct [17]. The final questionnaire encompassed three main parts: demographic data, knowledge and awareness of biosimilar medicines, and pharmacists’ attitudes toward biosimilar drugs.

### 2.4. Data Collection

Data collection was conducted through an anonymous online questionnaire created using Google Forms. The current research employed two non-probability sampling approaches, i.e., convenience and snowball sampling. Convenience sampling was initially utilized by the principal researcher, who visited accessible sites and invited pharmacists to participate by scanning a quick response (QR) code. Participants were also encouraged to share the study invitation with eligible colleagues, thereby initiating the snowball sampling method. Snowball sampling was further extended through social media platforms, i.e., WhatsApp and Telegram, where the study invitation along with eligibility criteria was disseminated with a request for participants to share the invitation with other eligible pharmacists. This facilitated access to a wider sample of pharmacists. The survey period spanned from January 2024 to February 2024. Participants were encouraged to participate by emphasizing the confidentiality of their involvement and underscoring the significance of the research to public health.

### 2.5. Ethical Considerations

King Khalid University’s Institutional Review Board (IRB) approved the study’s ethical considerations under the reference number (ECM#2024-104) on 16 January 2024. The study’s cover page contained the objectives, an anonymity statement, data confidentiality practices, and an informed consent statement. The present research was conducted in accordance with the Declaration of Helsinki.

### 2.6. Statistical Analysis

Data collected were cleaned, coded and analyzed using the Statistical Package for the Social Sciences (SPSS) version 21. Descriptive analysis involved frequency distributions and percentage calculations for all study variables.

The overall knowledge score was presented as the median and the 25–75 quartiles. The participants were classified into good and poor knowledge groups based on their total knowledge score, with the maximum possible score equal to 9. Those who scored above the median were categorized as having good knowledge of biosimilars, whereas participants who scored at or below the median were categorized as having poor knowledge [17].

Cross-tabulation was utilized to identify factors associated with pharmacists’ knowledge of biosimilar medicines, employing Pearson’s chi-square tests for significance and exact probability tests for small frequency distributions. Two-tailed statistical methods were employed, with an alpha level of 0.05 used to determine significance.

## 3. Results

A total of 298 pharmacists participated in the current study. Pharmacists’ ages ranged from 22 to 50 years, with a mean age of 32.5 ± 11.9 years. A total of 214 (71.8%) pharmacists were male, 154 (51.7%) held a Bachelor of Pharmacy (B.pharm), 84 (28.2%) had a Bachelor of Pharmacy (PharmD), and 60 (20.1%) had a postgraduate degree. As for experience, 89 (29.9%) had less than five years of experience, 91 (30.5%) had 5–10 years of experience, and 46 (15.4%) had more than 15 years of experience. A total of 164 (55%) worked in the private sector, and 134 (45%) worked in government hospitals. One hundred and thirty-five (45.3%) were community pharmacists, 124 (41.6%) worked in hospital pharmacies, and the remaining 13% were medical supply pharmacists, medical representatives, or pharmaceutical company pharmacists (Table 1).

Assessing the knowledge of pharmacists toward biosimilar medicines showed that 90.6% of the study pharmacists reported that they knew that “all FDA-approved biosimilar drugs are extensively evaluated to ensure patient confidence in their efficacy, safety, and quality”, 83.6% knew that “a biosimilar drug is an FDA-approved version of a reference biologic, manufactured after expiration of the biologic reference drug patent”, 76.2% knew that “similar variability exists between the biologic reference drug formulation lots and those of biosimilar drug formulation lots”, 68.8% knew that “a biosimilar drug has similar immunogenicity compared with the biologic reference drug”, and 59.7% knew that “a biosimilar drug does not differ in any clinically meaningful way when compared to the biologic reference drug, exhibiting comparable safety and efficacy”. Only 26.2% correctly reported that “biosimilar drugs are not interchangeable with the biological reference drugs”, 33.9% knew that “biosimilar drugs must not have an identical amino acid sequence to the biologic reference drug”, and 41.9% knew that “biosimilar drugs are not generics and do not have the same chemical structure as the biologic reference drug” (Table 2).

**Figure 1:**
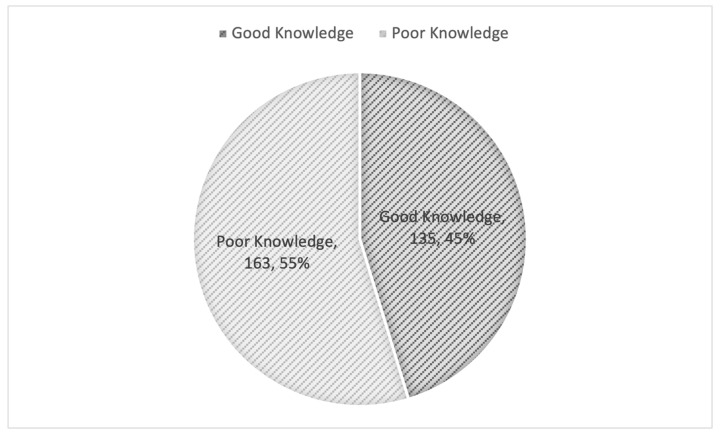
Overall Knowledge Level of Pharmacists in the Aseer Region Regarding Biosimilar Medicines.

As shown in Table 2, the median knowledge score among the study participants was 5 (5–6). Overall, a total of 135 (45.3%) demonstrated good knowledge of biosimilar medicines, while 163 (54.7%) exhibited poor knowledge (Figure 1).

Assessing the attitude of participants toward biosimilar medicines showed that 83.3% believed that biosimilar drugs enhance patients’ access to a broader range of treatment options, 73.2% expressed a willingness to substitute a biologic reference drug with a biosimilar drug if approved by the physician, 68.4% were in favor of dispensing biosimilar drugs, and 67.8% thought that patients should participate in deciding whether to use biosimilar drugs. Approximately 51.3% of pharmacists felt sufficiently trained to dispense and counsel patients on biosimilar drugs (Table 3).

The chi-square test examined the association between the socio-demographic characteristics of the pharmacists and their overall knowledge level (Table 4). Findings showed that there was a statistically significant association between years of experience and knowledge level of biosimilars (*p* = 0.049). Pharmacists with 11–15 years of experience had the highest proportion of good knowledge (56.9%), followed by those with more than 15 years (45.7%). In contrast, pharmacists with fewer than five years of experience showed the lowest proportion of good knowledge (38.2%). This indicates that greater professional experience is associated with a higher knowledge of biosimilar medicines. 

Field of work demonstrated a significant association with knowledge level (*p* = 0.048). Pharmacists working in medical supply had the highest proportion of good knowledge (52.4%), followed closely by those working as medical representatives or in pharmaceutical companies (50.0%). Community pharmacists had a lower proportion of good knowledge (43.0%). No other factors demonstrated a statistically significant association with pharmacists’ knowledge level. 

The chi-square test examined the association between pharmacists’ attitudes regarding biosimilars and their overall knowledge level (Table 5). There was a statistically significant association between pharmacists’ attitudes toward dispensing biosimilars and knowledge level. Pharmacists who were in favor of dispensing biosimilar drugs had the highest proportion of good knowledge (74.1%). This indicates that pharmacists with greater knowledge about biosimilar medicines were more likely to favor dispensing these medications, suggesting that increased knowledge may positively influence attitudes toward biosimilar use in practice. Other attitude items were not significantly associated with the pharmacists’ knowledge level. 

## 4. Discussion

The current study was set out to evaluate pharmacist’s knowledge of biosimilar medications in the Aseer region of Saudi Arabia, examine factors that may influence their knowledge level, and explore their attitudes toward these medicines. This focus aligns with the national health transformation initiative, which emphasizes value-based principles, wherein biosimilars are positioned as cost-effective options that can enhance treatment accessibility and support value-based care [20]. There is a growth in the biosimilar market in Saudi Arabia, and this trajectory is expected to continue in the next decade [20]. Within this evolving landscape, pharmacists play a central role in educating patients about biosimilar medications and guiding their appropriate use in clinical practice, thereby supporting a safe and cost-effective integration of biosimilars into healthcare delivery [17].

Existing literature on pharmacists’ knowledge and attitudes toward biosimilars in Saudi Arabia is limited, and used non-validated, self-developed questionnaires informed by the literature [12,21]. Internationally, the assessment tools employed across previously published studies were highly variable, and although many of them were validated, this variability limits the validity of cross-study comparisons and underscores the need for more standardized tools [1,6,8,17,18,22,23]. The current research used a validated tool adapted from the instrument developed by Abusara et al. (2023), which demonstrated an acceptable internal consistency with Cronbach’s alpha above 0.7 [17]. Our study addresses a key gap in the literature by providing more reliable, comparable evidence on pharmacist’ knowledge and attitudes of biosimilars within the Saudi context. This contribution may help in strengthening the evidence base needed to inform future practice and policy. 

In our research, pharmacists showed a median knowledge score of 5 (5–6), and this closely aligns with Abusara et al.’s (2023) findings, who also reported a median score of 5 (4–6) among Jordanian pharmacists working in various fields [17]. However, variation in the pattern of correct responses was observed between the two studies. The discrepancies observed between the current research and the previous Jordanian study could be attributed to variation in pharmacists’ exposure to biosimilar related information across countries, whether through educational curricula or professional development opportunities.

In the present study, the highest knowledge level was observed among items related to the extensive FDA evaluation of biosimilars (90.6%), regulatory definitions (83.6%), and the manufacturing variability (76.2%). Conversely, Abusara and colleagues findings [17] showed that the highest knowledge for items related to molecular similarity and clinical equivalence, with correct responses exceeding 90%. It is worth noting that only 26.2% of pharmacist in the current study correctly identified that biosimilars are not generics and not interchangeable with reference biologics, while a substantial higher correct responses was observed for the same item in the Jordanian study (76.5%) [17]. This finding is important because, unlike traditional generic medicines that can be substituted at the pharmacy level without prescriber approval, biosimilars generally require clinician intervention for substitution, unless they have met additional FDA requirements to receive an interchangeable designation [13,14,15]. Knowledge gaps highlighted in the current study underscores the need for targeted interventions to strengthen pharmacist knowledge of biosimilars, particularly regarding biosimilars interchangeability and regulatory distinctions from traditional generic medicines. This could support more appropriate utilization of biosimilars and contribute to improved patient outcomes. Higher biosimilar knowledge in the present study was linked to pharmacists’ experience and field of work. Those working in medical supply and industrial pharmacy demonstrated the greatest understanding. 

In agreement with findings from previous research, pharmacists in the current study demonstrated generally favorable attitudes toward biosimilar medicines [3,8,10,12,18]. Most participants acknowledged the role of biosimilars in improving treatment access and expressing support for their dispensing and prescriber-approved substitution. Participants also showed openness to involving patients in decisions about biosimilar use. Despite these positive attitudes, only half of the participants felt adequately trained to dispense biosimilars. This highlights the need for targeted educational initiatives to enhance pharmacists’ confidence and readiness in this area. Findings from the present study also showed an association between favoring the dispensing of biosimilars and having good knowledge about them. This suggests that enhancing pharmacists’ knowledge of biosimilars may help strengthen their support for their dispensing in practice.

## 5. Limitations of the Study

Although this was a well-designed study using a validated questionnaire, the findings are subject to several limitations. Firstly, the use of non-probability sampling methods may have limited the representative of the sample and introduced selection bias, as recruitment depended on accessibility and social networks. A gender imbalance was also noted, with females underrepresented in the current sample. This might limit the representativeness of the current study findings to the wider population. Secondly, the use of a self-administered questionnaire may have introduced repones bias; such bias cannot be ruled out due to the self-reported nature of the data. Finaly, the achieved sample size was 12 participants below the minimum recommended size, which may have affected the statistical power.

## 6. Conclusions and Recommendations

The current study showed that pharmacists’ knowledge of biosimilars remains limited, particularly in areas related to the policy and regulatory requirements for interchangeability and substitution. More experienced pharmacists and those working in industry-related sectors demonstrated greater knowledge. The overall attitudes toward biosimilars were positive, with a clear association observed between higher knowledge and greater support for dispensing biosimilars. Therefore, strengthening pharmacists’ understanding of biosimilars may enhance their confidence in dispensing these products in practice. It is recommended that targeted educational initiatives, continuing professional development opportunities, and enhanced curricular content be implemented to address key knowledge gaps. Additionally, further legislation is needed to guide the dispensing of biosimilars.

## Figures and Tables

**Table 1:** Socio-demographic characteristics of the study participants (*n* = 298).

Demographic Data	N (%)
Age in years	
22–30	108 (36.2%)
31–40	146 (49.0%)
41–50	44 (14.8%)
Gender	
Male	214 (71.8%)
Female	84 (28.2%)
Academic Level	
Bachelor B.pharm	154 (51.7%)
Bachelor PharmD	84 (28.2%)
Postgraduate	60 (20.1%)
Experience years	
<5 years	89 (29.9%)
5–10 years	91 (30.5%)
11–15 years	72 (24.2%)
>15 years	46 (15.4%)
Place of work	
Public sector	134 (45.0%)
Private sector	164 (55.0%)
Field of work	
Community Pharmacies	135 (45.3%)
Hospital pharmacies (Inpatients/outpatients)	124 (41.6%)
Medical supply	21 (7.0%)
Medical Representative or pharmaceutical company pharmacist	18 (6.0%)

**Table 2:** Knowledge of Pharmacists in the Aseer Region Toward Biosimilar Medicines.

Knowledge Items	N (%)
A biosimilar drug has no clinically meaningful differences (similar safety and efficacy) compared to the biological reference drug.	
True ^**#**^	178 (59.7%)
False	120 (40.3%)
A biosimilar has similar immunogenicity compared with the biologic reference drug.	
True ^**#**^	205 (68.8%)
False	93 (31.2%)
A biosimilar drug is a generic, has same chemical structure, as the biologic reference drug.	
True	173 (58.1%)
False ^**#**^	125 (41.9%)
A biosimilar drug must have the exact amino acid sequence as the biologic reference drug.	
True	197 (66.1%)
False ^**#**^	101 (33.9%)
A biosimilar drug is interchangeable with the biologic reference drugs.	
True	220 (73.8%)
False ^**#**^	78 (26.2%)
There is similar variability between the biologic reference drug formulation lots as there is a variability in biosimilar drug formulation lots.	
True ^**#**^	227 (76.2%)
False	71 (23.8%)
All FDA-approved biosimilar drugs undergo an extensive assessment to make sure that patients can trust their efficacy, safety, and quality.	
True ^**#**^	270 (90.6%)
False	28 (9.4%)
A biosimilar drug manufacturing cost is higher than that of the biologic reference drug.	
True	113 (37.9%)
False ^**#**^	185 (62.1%)
A biosimilar drug is an FDA-approved version of a biologic reference drug that is manufactured after the expiry of the biologic reference drugs patent.	
True ^**#**^	249 (83.6%)
False	49 (16.4%)
Knowledge score Median (IQR)	5.00 (5.00–6.00)

^#^: Correct answer; IQR: Interquartile Range.

**Table 3:** Pharmacists’ Attitude Toward Biosimilar Medicines.

Attitude	Strongly Disagree	Disagree	Neutral	Agree	Strongly Agree
	N (%)	N (%)	N (%)	N (%)	N (%)
I am in favor of dispensing biosimilar drugs	6 (2.0%)	17 (5.7%)	71 (23.8%)	144 (48.3%)	60 (20.1%)
I think that biosimilar drugs increase patients’ access to a variety of treatment options	1 (0.3%)	7 (2.3%)	42 (14.1%)	159 (53.4%)	89 (29.9%)
I am willing to substitute a biologic reference drug with a biosimilar drug if the physician approved it	7 (2.3%)	16 (5.4%)	57 (19.1%)	142 (47.7%)	76 (25.5%)
I feel that I am trained enough to dispense and counsel patients of biosimilar drugs	18 (6.0%)	48 (16.1%)	79 (26.5%)	104 (34.9%)	49 (16.4%)
I think that patients should participate in taking decision to use biosimilar drugs	10 (3.4%)	22 (7.4%)	64 (21.5%)	131 (44.0%)	71 (23.8%)

**Table 4:** Factors Associated with the Knowledge Level of Pharmacists Toward Biosimilar Medicines.

Factors	Overall Knowledge Level		*p*-Value
	Poor	Good	
	N (%)	N (%)	
Age in years			0.186
22–30	65 (60.2%)	43 (39.8%)	
31–40	72 (49.3%)	74 (50.7%)	
41–50	26 (59.1%)	18 (40.9%)	
Gender			0.807
Male	118 (55.1%)	96 (44.9%)	
Female	45 (53.6%)	39 (46.4%)	
Academic Level			0.476
Bachelor B.pharm	79 (51.3%)	75 (48.7%)	
Bachelor PharmD	49 (58.3%)	35 (41.7%)	
Postgraduate	35 (58.3%)	25 (41.7%)	
Experience years			0.049 *
<5 years	55 (61.8%)	34 (38.2%)	
5–10 years	52 (57.1%)	39 (42.9%)	
11–15 years	31 (43.1%)	41 (56.9%)	
>15 years	25 (54.3%)	21 (45.7%)	
Work sector			0.869
Public sector	74 (55.2%)	60 (44.8%)	
Private sector	89 (54.3%)	75 (45.7%)	
Field of work			0.048 * ^
Community Pharmacies	77 (57.0%)	58 (43.0%)	
Hospital pharmacies (Inpatients/outpatients)	67 (54.0%)	57 (46.0%)	
Medical Representative or pharmaceutical company pharmacist	9 (50.0%)	9 (50.0%)	
Medical supply	10 (47.6%)	11 (52.4%)	

*p*: Pearson X^2^ test; ^: Exact probability test; * *p* < 0.05 (significant).

**Table 5:** Association between pharmacists’ knowledge and their attitudes regarding biosimilar drugs.

Attitude	Overall Knowledge Level						*p*-Value
	Poor			Good			
	Disagree	Neutral	Agree	Disagree	Neutral	Agree	
	N (%)	N (%)	N (%)	N (%)	N (%)	N (%)	
I am in favor of dispensing biosimilar drugs	13 (8.0%)	46 (28.2%)	104 (63.8%)	10 (7.4%)	25 (18.5%)	100 (74.1%)	0.049 *
I think that biosimilar drugs increase patients’ access to a variety of treatment options	6 (3.7%)	22 (13.5%)	135 (82.8%)	2 (1.5%)	20 (14.8%)	113 (83.7%)	0.490
I am willing to substitute a biologic reference drug with a biosimilar drug if the physician approves it	12 (7.4%)	35 (21.5%)	116 (71.2%)	11 (8.1%)	22 (16.3%)	102 (75.6%)	0.525 ^
I feel that I am trained enough to dispense and counsel patients about biosimilar drugs	32 (19.6%)	46 (28.2%)	85 (52.1%)	34 (25.2%)	33 (24.4%)	68 (50.4%)	0.479 ^
I think that patients should participate in deciding to use biosimilar drugs	18 (11.0%)	33 (20.2%)	112 (68.7%)	14 (10.4%)	31 (23.0%)	90 (66.7%)	0.848

*p*: Pearson X^2^ test; ^: Exact probability test; * *p* < 0.05 (significant).

## Data Availability

The original contributions presented in the study are included in the article; further inquiries can be directed to the corresponding author.
